# The Distribution of Dissolved Iron in the West Atlantic Ocean

**DOI:** 10.1371/journal.pone.0101323

**Published:** 2014-06-30

**Authors:** Micha J. A. Rijkenberg, Rob Middag, Patrick Laan, Loes J. A. Gerringa, Hendrik M. van Aken, Véronique Schoemann, Jeroen T. M. de Jong, Hein J. W. de Baar

**Affiliations:** 1 Department of Biological Oceanography, Royal Netherlands Institute for Sea Research, Den Burg, the Netherlands; 2 Department of Chemistry, University of Otago, Dunedin, New Zealand; 3 Department of Ocean Ecosystems, University of Groningen, Groningen, the Netherlands; CINVESTAV-IPN, Mexico

## Abstract

Iron (Fe) is an essential trace element for marine life. Extremely low Fe concentrations limit primary production and nitrogen fixation in large parts of the oceans and consequently influence ocean ecosystem functioning. The importance of Fe for ocean ecosystems makes Fe one of the core chemical trace elements in the international GEOTRACES program. Despite the recognized importance of Fe, our present knowledge of its supply and biogeochemical cycle has been limited by mostly fragmentary datasets. Here, we present highly accurate dissolved Fe (DFe) values measured at an unprecedented high intensity (1407 samples) along the longest full ocean depth transect (17500 kilometers) covering the entire western Atlantic Ocean. DFe measurements along this transect unveiled details about the supply and cycling of Fe. External sources of Fe identified included off-shelf and river supply, hydrothermal vents and aeolian dust. Nevertheless, vertical processes such as the recycling of Fe resulting from the remineralization of sinking organic matter and the removal of Fe by scavenging still dominated the distribution of DFe. In the northern West Atlantic Ocean, Fe recycling and lateral transport from the eastern tropical North Atlantic Oxygen Minimum Zone (OMZ) dominated the DFe-distribution. Finally, our measurements showed that the North Atlantic Deep Water (NADW), the major driver of the so-called ocean conveyor belt, contains excess DFe relative to phosphate after full biological utilization and is therefore an important source of Fe for biological production in the global ocean.

## Introduction

In the ancient oxygen free and sulphidic ocean, high abundances of first row transition metals led to their implementation in many metabolic functions during biological evolution [1], [2]. Among the six trace metals Mn, Fe, Co, Ni, Cu and Zn essential for every living cell and organism, Fe is by far the most important. Protein-bound Fe acts as vital electron mediator for several metabolic processes like nitrogen assimilation, N_2_-fixation, photosynthetic and respiratory electron transport and the removal of reactive oxygen species [3]. However, the subsequent evolution of biogenic oxygen (O_2_) by photosynthesis in the oceans [4], [5] favored the Fe(III) redox state with a very low solubility [6] leading to massive precipitation of iron-oxides, and thus to the removal of almost all dissolved Fe (DFe) from the oceans [7], [8]. Consequently, low concentrations of DFe in the modern ocean affect marine ecosystems and the related carbon cycle in large parts of the world’s oceans [9], [10], [11], [12].

Measurement of extremely low concentrations of DFe (DFe, <0.2 µm filter pore size), as found in the open ocean, has a high risk of contamination and requires ultraclean techniques throughout the complete procedure of sampling, sample processing and final analysis [13]. Therefore measurements of DFe are relatively scarce and fragmentary often making interpretation difficult and speculative, and limiting progress in our knowledge of the biogeochemical cycling of DFe. The importance of Fe for marine ecosystems makes Fe perhaps the most wanted chemical trace element for study in the international GEOTRACES program (www.geotraces.org) that aims to measure the distribution of important trace elements and isotopes in the global oceans [14].

The distribution of DFe depends on its chemistry, sinks, and internal cycling, sources and transport. The chemistry of Fe in modern oxygenated oceans is characterized by its very low solubility (0.1–0.2 nM) [6]. However, complexation of Fe by organic Fe-binding ligands increases the solubility of Fe [15], [16]. The excess of free organic Fe-binding ligands often present in the oceans plays an essential role in the solubilization and stabilization of Fe entering the ocean from external sources [17]. Fe-binding organic ligands exist in the colloidal (0.02–0.2 µm) as well as the truly dissolved phase (<0.02 µm) [18], [19]. Colloidal Fe(III) is vulnerable for removal by adsorption onto large settling (mostly biogenic) particles falling down from surface waters. This scavenging of Fe is the main removal mechanism of Fe from the oceans. Photo-reduction of this colloidal Fe, and its interaction with organic Fe-binding ligands may therefore be an important process retaining DFe in the surface waters [20], [21], [22], [23] and in increasing its biological availability [24], [25], [26]. Thus organic complexation of Fe plays an essential role in determining the distribution of DFe [27].

Biological uptake of Fe, as well as adsorption onto biological matter in ocean surface waters, results in the transport of DFe from the surface ocean to deeper waters (200–1000 m) by the settling of biogenic debris and subsequent remineralization at depth. Below highly productive oceanic regions, the combination of enhanced oxygen consumption during organic matter respiration and weak ocean ventilation leads to the formation of Oxygen Minimum Zones (OMZ) at these intermediate depths [28]. In an OMZ, DFe concentrations are enhanced and stabilized due to the simultaneous release of organic Fe-binding ligands [29], [30], [31] and enhanced Fe(II) concentrations that may exist under low oxygen conditions [30], [32], [33].

The most important external source of DFe to the remote surface ocean is aeolian dust. The western North Atlantic Ocean receives about 43% of global oceanic dust inputs. This dust originates from the Sahara and Sahel regions [34], [35]. The western South Atlantic Ocean receives less dust, mainly originating from Patagonia [36]. River input is another important source of DFe to the surface ocean. The overall DFe input from rivers into the world’s oceans is estimated to be 26 10^9^ mol y^−1^ [37]. The majority of DFe in river water exists as small colloid particles [38], [39], [40] which flocculate and are removed from solution upon mixing with seawater [41]. Only the organically complexed DFe is flushed from the estuary into the ocean [42], [43].

Several studies have shown that continental shelves are also important sources of DFe to the open ocean [44]. Oxidation of organic material in shelf sediments may lead to suboxic or anoxic conditions with reductive Fe-oxyhydroxide dissolution and subsequent release of DFe to the overlying water column [45]. Alternatively, upwelling of deep waters enhanced in DFe, or release of Fe from upwelled biogenic and lithogenic particles along a shelf, may explain the gradient of surface DFe concentration with distance from the continental shelf [46]. Horizontal transport of dissolved and particulate iron derived from continental shelf regions determined DFe distributions off the Californian coast [44], in the subarctic North Pacific [47], [48], in the vicinity of the Crozet and Kerguelen Islands [49], [50], offshore of the Antarctic Peninsula [51], [52] and in the polynyas of Pine Island Bay and the Amundsen Sea in the Southern Ocean [53].

Recent observations show that hydrothermal vents are a more significant source of DFe to the deep ocean than previously realized [54], [55], [56], [57], [58]. Model simulations show that deep hydrothermal input enhances surface water concentrations of DFe with an estimated 3% of the global mean [59]. However, this modeled estimate may be an underestimation as hydrothermal vents along the Mid Atlantic Ridge appear to contribute a significant hydrothermal Fe flux to the South Atlantic Basin [60].

Revisiting the 1972 West Atlantic GEOSECS section, this study covers the DFe distribution in the western Atlantic Ocean following the southward transport of the North Atlantic Deep Water (NADW), which is the major driver of the so-called ocean conveyor belt [61], [62]. We present in this study the longest full ocean depth transect of highly accurate DFe values measured along the entire western Atlantic Ocean. We explain the distribution of DFe with respect to Fe sources, sinks and internal processes occurring in the Western Atlantic Ocean (GEOTRACES GA02 section).

## Materials and Methods

**Figure 1:**
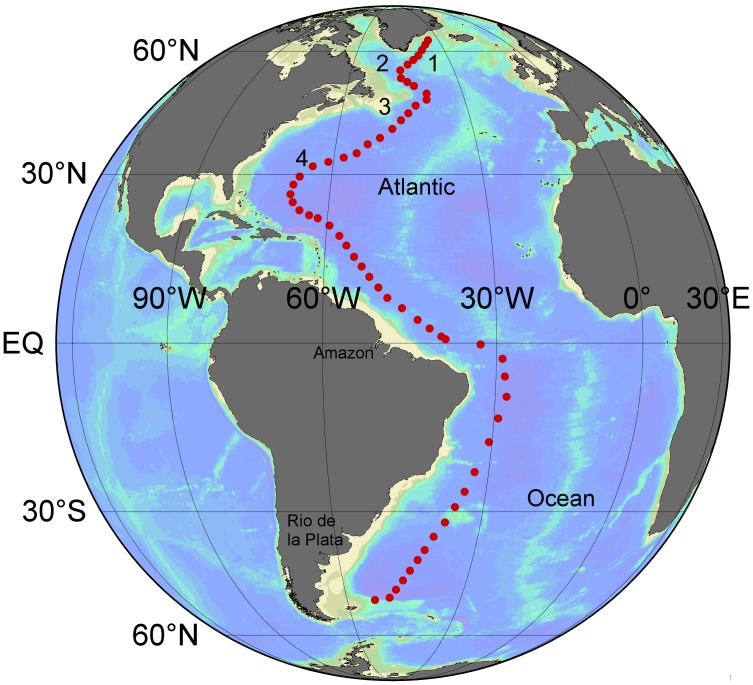
With 60 full depth stations over 17500*Pelagia* and 74JC057 on the RRS *James Cook* covered the complete West Atlantic Ocean GEOTRACES GA02 transect. Numbers show the locations 1) Irminger Sea, 2) Labrador Sea, 3) the Grand Banks, and 4) Bermuda.

The 60 stations (1407 DFe values) covering the whole west Atlantic Ocean GEOTRACES GA02 transect from Iceland to the tip of South America were completed in four cruises. Leg 1 from Iceland to Bermuda on RV *Pelagia* (64PE319) was sailed in April/May 2010, leg 2 from Bermuda to Fortaleza (Brazil) on RV *Pelagia* (64PE321) was sailed in June/July 2013, and leg 3 from Punta Arenas (Chile) to Las Palmas (Canary Islands, Spain) on the RRS *James Cook* (74JC057) was sailed in March/April 2011. An additional cruise with RV *Pelagia* from Iceland to Texel was sailed in July/August 2012 to sample five northern stations that were missed in 2010 due to severe storms (Fig. 1). Most stations were in international waters thus no specific permissions were required for activities at these stations. We had permission from the Danish Ministry of Foreign Affairs to sample at station 3 (57°12.65′N, 41°35.95′W) during cruise 64PE358.

Samples were taken using 24 ultra trace-metal clean PVDF samplers of 24 L each mounted on an all titanium frame with a SEABIRD 911 CTD system and deployed with a Kevlar hydrowire [13], [63]. After deployment, the complete “ultraclean CTD” was immediately placed in an ISO Class 6 clean room container, where samples for dissolved metals were filtered directly from the PVDF samplers over<0.2 µm Sartobran 300 cartridges (Sartorius) under pressure of filtered N_2_ applied via the top-connector of the PVDF sampler. Samples for dissolved metals were acidified to a pH of 1.8 using a final concentration of 0.024 M ultraclean Seastar Base-line HCl (Seastar Chemicals).

DFe was measured on board using flow injection analysis (FIA) based on luminol chemiluminescence [58]. Briefly, to measure DFe, 60 µL of 10 mM H_2_O_2_ (Suprapure, Merck 30%) was added to 60 ml sample to ensure the oxidation of any Fe(II) in the sample at least 12 h prior to analysis [64]. Next, the acidified sample was pre-concentrated in-line onto a column of immobilized AF-Chelate-650 M resin (TosoHaas, Germany). After rinsing with de-ionized water (18.2 MΩ cm, Millipore) to remove interfering salts, the Fe was eluted from the column with 0.4 M HCl (Suprapure, Merck 30%). Chemiluminescence was induced by mixing the eluent with a 0.96 M ammonium hydroxide (Suprapur, 25% Merck), 0.3 M hydrogen peroxide (Suprapure, Merck 30%), 0.3 mM luminol (Aldrich), and 0.7 mM triethylenetetramine (Sigma). All solutions were prepared with de-ionized water (18.2 MΩ cm, Millipore). The chemiluminescence was detected with a Hamamatsu HC135 Photon counter. A five-point calibration and blank determination were made daily. The blank was determined as the intercept of the signals of increasing pre-concentration times (5, 10, 15 seconds) of the seawater used for the calibration. The analytical blank was on average 0.013±0.012 nM DFe (n = 66) and the average detection limit (defined as 3σ of the blank pre-concentrated for 5 seconds) was 0.012±0.012 nM DFe (n = 66). The Fe added by the Seastar acid (maximum ∼0.4 pM) was neglected.

Dissolved Aluminium (DAl) was measured on board using flow injection analysis (FIA) based on a lumogallion fluorometric method [65]. The dissolved Manganese (DMn) was measured on board using FIA based on luminol chemiluminescence [66].

Measurements of samples from the SAFe/GEOTRACES intercomparison-program ensured quality control (Table 1). The high quality of the data was demonstrated by the good agreement between field measurements and SAFe/GEOTRACES reference samples, by a good internal consistency (see figures with correlations) and by the excellent agreement between the NIOZ GEOTRACES data and the US GEOTRACES data on the BATS cross over station (32°N, 64°W).

**Table 1:** The values of SAFe and GEOTRACES reference samples for DFe, DAl and DMn measured during the cruises 64PE319, 64PE321 & 64PE358 in the western North Atlantic Ocean and cruise 74JC057 in the western South Atlantic Ocean.

Measured	Sample	Bottle #	element	Value	n	units
North Atlantic	SAFe D2	389	Fe	0.97±0.07	7	nmol/L
North Atlantic	SAFe D2	499	Fe	0.91±0.06	9	nmol/L
North Atlantic	SAFe D2	504	Fe	0.90±0.05	5	nmol/L
South Atlantic	SAFe D2	246	Fe	0.91±0.05	5	nmol/L
South Atlantic	SAFe D2	449	Fe	0.99±0.01	3	nmol/L
North Atlantic	GS	100	Al	28.04±0.38	9	nmol/L
North Atlantic	GD	147	Al	18.04±0.44	11	nmol/L
North Atlantic	SAFe S	91	Al	1.71±0.03	3	nmol/L
South Atlantic	GS	54	Al	28.09±0.13	2	nmol/L
South Atlantic	GS	100	Al	28.00	1	nmol/L
South Atlantic	GD	38	Al	17.90±0.18	9	nmol/L
South Atlantic	GD	146	Al	17.61	1	nmol/L
North Atlantic	SAFe D2	404	Mn	0.34±0.01	9	nmol/L
North Atlantic	SAFe D2	58	Mn	0.33±0.01	6	nmol/L
North Atlantic	GS	52	Mn	1.50±0.02	2	nmol/L
North Atlantic	GS	55	Mn	1.75±<0.01	2	nmol/L
North Atlantic	GS	100	Mn	1.53±0.02	3	nmol/L
North Atlantic	GD	146	Mn	0.17±<0.01	2	nmol/L
North Atlantic	GD	147	Mn	0.18±<0.01	2	nmol/L
South Atlantic	SAFe D2	58	Mn	0.34±<0.01	3	nmol/L
South Atlantic	SAFe D2	575	Mn	0.35±0.01	6	nmol/L
South Atlantic	GS	54	Mn	1.48±0.02	5	nmol/L
South Atlantic	GD	38	Mn	0.18	1	nmol/L
Intercalibration consensus values as per May 2013:						
May-2013	SAFe D2		Fe	0.933±0.023		nmol/Kg
May-2013	SAFe D2		Mn	0.35±0.05		nmol/Kg
May-2013	SAFe S		Al	1.67±0.10		nmol/Kg
May-2013	GS		Al	27.5±0.2		nmol/Kg
May-2013	GD		Al	17.7±0.2		nmol/Kg
May-2013	GS		Mn	1.50±0.11		nmol/Kg
May-2013	GD		Mn	0.21±0.03		nmol/Kg

Also included are the SAFe and GEOTRACES intercalibration consensus values (http://www.geotraces.org/science/intercalibration). GS is a GEOTRACES surface reference sample and GD is a GEOTRACES deep reference sample.

Oxygen was measured using a Seabird SBE 43 oxygen sensor on the titanium CTD frame. The oxygen sensor was calibrated using Winkler titrations on discretely collected samples from both the titanium CTD frame, as well as the regular CTD rosette. Salinities were determined using a SBE3plus thermometer and a SBE4 conductivity sensor on the titanium frame. The salinity of discrete seawater samples were analyzed on board with a Guildline 8400B Autosal using an OSIL standard water batch P149 to calibrate the salinity measurements of the CTD sensors. The temperature sensor was calibrated against a SBE-35 reference thermometer. Nitrate, phosphate and silicate were determined colorimetrically [67] on a Bran en Luebbe trAAcs 800 Auto-analyzer. Unfiltered samples were taken from each bottle of each CTD and analyzed on board within 8–12 hours.

## Results and Discussion

### Hydrography

**Figure 2:**
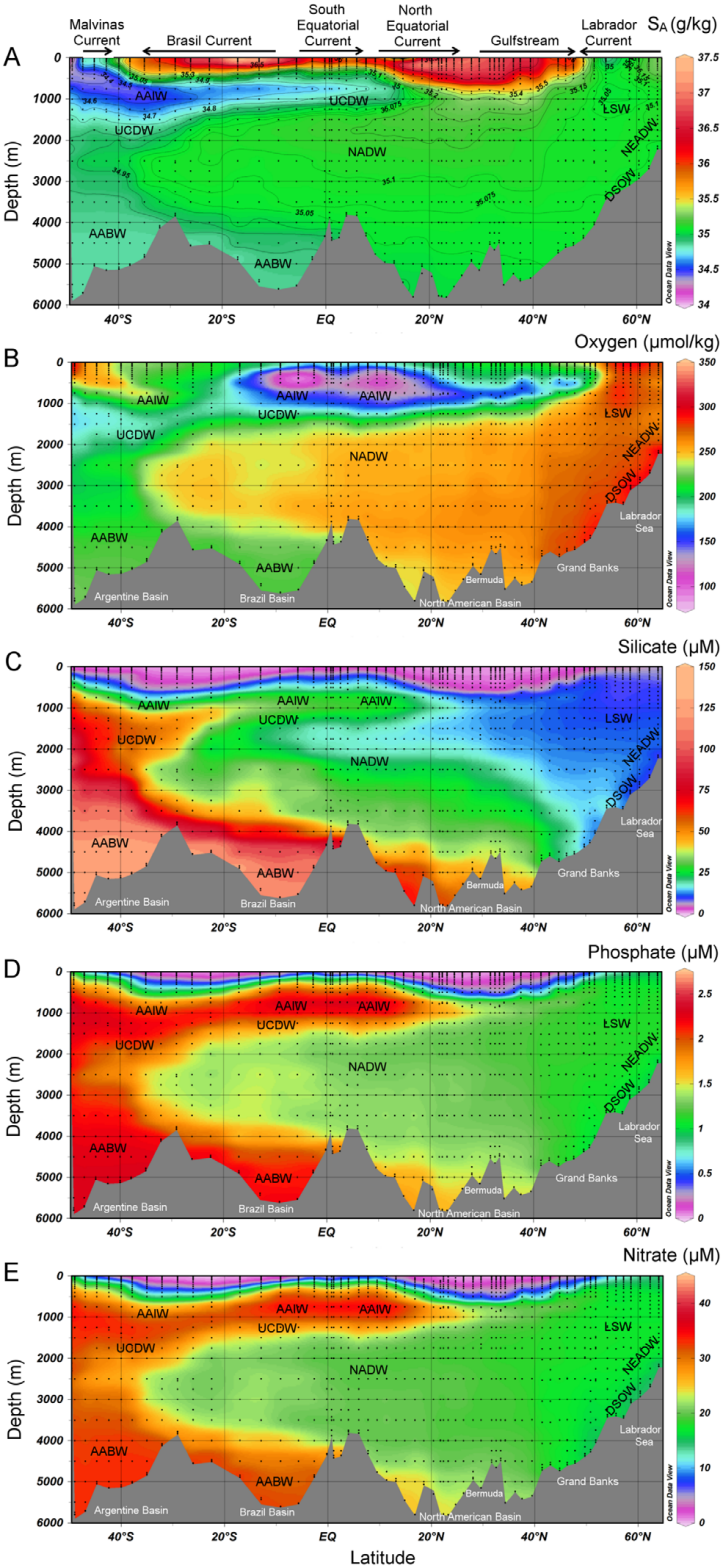
Section plots of A) the absolute salinity (S_A_), B) oxygen, C) silicate, D) phosphate, and E) nitrate as measured during the GEOTRACES cruise 64PE319, 64PE321, 64PE358 and 74JC057 in the west Atlantic Ocean. Water masses indicated are Labrador Sea Water (LSW), North East Atlantic Deep Water (NEADW), Denmark Strait Overflow Water (DSOW), North Atlantic Deep Water (NADW), AntArctic Intermediate Water (AAIW), Upper Circumpolar Deep Water (UCDW), and AntArctic Bottom Water (AABW). Section plots of the salinity, oxygen and nutrients from the GEOTRACES GA02 transect without the results of 64PE358 have been published before [72], [97].

Section plots of absolute salinity, oxygen and nutrient data provided a general overview of the water masses encountered in the West Atlantic Ocean (Fig. 2). Close to the northern end of our transect, the Denmark Strait between Iceland and Greenland is an important connection between the Nordic Seas and the Atlantic Ocean. Near the sea surface, cold and fresh polar waters flow into the Irminger Sea (Fig. 1), in the East Greenland current and in the East Greenland shelf current. In the bottom layer (>∼1800 m depth), the cold Denmark Strait Overflow Water (DSOW) flows across the topographic sill [62]. Between the surface and 1800 m, the salinity decreased towards the Labrador Sea indicating Labrador Sea Water (LSW) between ∼600–1100 m and North East Atlantic Deep water (NEADW) between ∼1100–1800 m. The NEADW originates from the inflow of cold Iceland-Scotland Overflow Water (ISOW) flowing from the Norwegian Sea, between Iceland and Scotland, into the Iceland Basin. The LSW, NEADW, and DSOW all contribute to the formation of the North Atlantic Deep Water (NADW) that flows to the Southern Ocean [62].

In the surface waters around the Grand Banks, relatively cold and fresh waters from the North encounter the warmer and more saline waters of the Gulfstream. At depth, the LSW can be divided in an upper-LSW (uLSW) between the seawater densities 27.68 and 27.74 kg m^−3^ and the LSW between 27.74 and 27.80 kg m^−3^ [68]. The uLWS varied in depth from 350–1200 m at 54°N to 950–1300 m at 44°N and the LSW varied in depth from 1200–1950 m at 54°N and 1300–2300 m at 44°N. Near bottom oxygen maxima in the oxygen profiles around the Grand Banks indicated a near bottom layer of DSOW topped by traces of intruding Antarctic Bottom Water (AABW), as indicated by a maximum in the concentrations of silicate (Fig. 2B, C).

In the upper 1200 m between the Grand Banks and the Bermuda Rise, we found the eastward flowing water of the northern part of the subtropical gyre transporting water from the Gulfstream to the Azores Current and the North Atlantic Current. Only at a station at 37.5°N and at a depth of ∼1000 m did a maximum in salinity and DAl indicate the presence of a component of Mediterranean Sea Outflow Water (MSOW). From about 40°N southward below 2000 m depth relative oxygen maxima with a minimum in between represented the upper, middle and lower NADW (NADWu (2000–2500 m), NADWm (2500–3000 m), and NADWl (center ∼4000 m), respectively) together forming the NADW complex. The NADWu, NADWm and NADWl originated from the LSW, NEADW, and DSOW respectively [62]. High silicate concentrations and a lower salinity indicated AABW below the NADW.

In the southern half of the North Atlantic Ocean, the surface waters in the upper 1200 m of the subtropical gyre flow in a westward direction feeding the Gulfstream. At ∼900 m depth a salinity minimum coincided with a silicate maximum indicating Antarctic Intermediate Water (AAIW) (Fig. 2A, C). Below the AAIW, the vertical dissolved oxygen distribution showed the NADW complex. The presence of AABW was indicated by a slight decrease in salinity and temperature that coincided with an increase in silicate in the deepest 1000 m.

To the South of the subtropical gyre between 2.5° and 17.5°N we encountered the Amazon plume in the upper 50 m. North of 10°N, we encountered the westward flowing North Equatorial Current (NEC) in the upper 1000 m [69]. To the South of 7.5°N, we encountered the north-westward flowing North Brazil Current (NBC) [70]. Here, in the thermocline between 9.5–18.5°N, the more saline North Atlantic Central Water (NACW) was replaced by the less saline South Atlantic Central Water (SACW). Between 750–1000 m, the AAIW was characterized by a minimum in salinity and a maximum in silicate concentration. Below the AAIW lays the NADW complex, and below 4500 m a lower salinity coinciding with an increase in silicate concentrations indicated the AABW.

In the South Atlantic Ocean, the surface SACW can be recognized in a property-property plot of potential temperature against salinity as a straight line between 5°C, 34.3 and 20°C, 36.0 [71]. The AAIW could be recognized by low salinities of around 34 and appeared around 50°S flowing at a depth between about 500–1000 m northwards (Fig. 2). The North Atlantic Deep Water (NADW) with salinities around 35 flows at a depth between 1200–3900 m southwards until 25°S after which it starts to flow eastwards [62], however, components of the NADW could still be found along our transect until 45°S [71]. Between the equator and 30°S, low silicate concentrations and a salinity maximum within the NADW indicated the NADWu while slight differences in oxygen still allowed the distinction of the NADWm and NADWl in this study. As we could not identify the separate layers of the NADW complex between 30°S and 45°S we therefore refer here to NADW. The NADW penetrated the Circumpolar Deep Water (CDW) entering from the Drake Passage dividing the CDW in an Upper and Lower CDW. The Upper Circumpolar Deep Water (UCDW) flowed below the AAIW northwards (Fig. 2). Along our transect between 49°S and ∼25°S, the UCDW was indicated by an oxygen minimum while the UCDW further north was identified by high silicate and phosphate maxima (Fig. 2D). The UCDW’s most northern reaches are found near the equator [71]. The AABW, characterized by low salinities and high silicate concentrations, flowed below 4000 m northwards.

### The distribution of DFe

**Figure 3:**
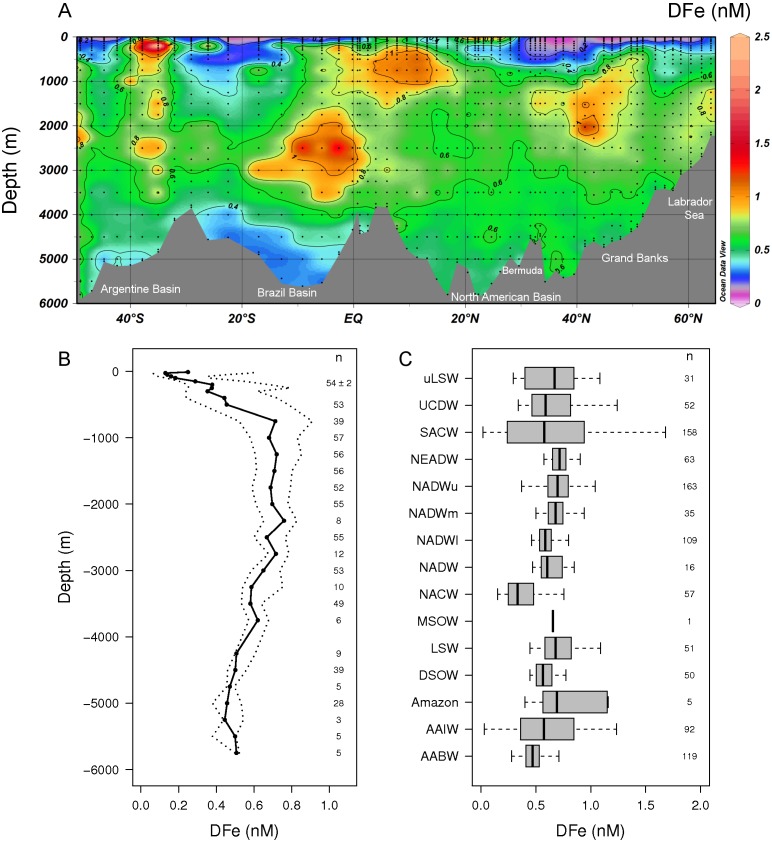
Distribution of DFe in the western Atlantic Ocean. A) 17500 km long full depth section plot of DFe (nM) in the western Atlantic Ocean measured on board ship in May–June 2010 and March–April 2011. B) Summary of the vertical distribution of all DFe concentrations measured during this study in the western Atlantic Ocean showing the median DFe (solid line with dots). The interquartile range defined as the range around the median containing 50% of the data is given between the two dotted lines. The number of samples per depth region (n) is shown in the plot. The 10 sampling depths in the upper water column have each on average 54±2 data points. C) A boxplot of the DFe concentrations in each of the water masses as encountered along our transect in alphabetical order Antarctic Bottom Water (AABW), Antarctic Intermediate Water (AAIW), the Amazon plume, Denmark Strait Overflow Water (DSOW), Labrador Sea Water (LSW), Mediterranean Sea Outflow Water (MSOW), North Atlantic Central Water (NACW), North Atlantic Deep Water (NADW), the lower, middle and upper NADW (NADWl, NADWm, NADWu), the North East Atlantic Deep Water (NEADW), the South Atlantic Central Water (SACW), the Upper Circumpolar Deep Water (UCDW) and the upper LSW (uLSW). Median values are indicated by a vertical line within the box, the box represents the interquartile range, and the whiskers extend to the 5th and 95th percentile values. Outliers, defined as data points more than 1.5 times the interquartile range above the third quartile and points more than 1.5 times the interquartile range below the first quartile, are not shown.

The systematic collection of 1407 highly accurate DFe measurements distributed over the full ocean depth along a transect between 65°N and 50°S in the Western Atlantic Ocean showed DFe concentrations ranging between 0.018 to 6.1 nM with an overall mean of 0.55±0.36 nM (n = 1407, s.d.) (Fig. 3A). At a first glance the distribution of DFe did not appear to follow the main West Atlantic water mass structure as for example the nutrients nitrate, phosphate and silicate (Fig. 2C, D, E) and the trace metal dissolved Aluminium (DAl) [72]. The distribution of DFe was characterized by external sources that increased, and by other processes that either increased or decreased the DFe concentration with respect to the background concentration. Nevertheless, taking all measurements together, the median DFe concentration (Fig. 3B) showed a hybrid-type depth distribution typical for DFe [72]. In general, and as found in our study, hybrid-type depth distributions are nutrient-like in the upper 1000 m, where biological uptake of Fe results in low concentrations in the surface, followed by an increase in DFe with depth due to remineralization of sinking organic matter resulting in the release of DFe. In the West Atlantic Ocean, between 1000 and 2000 m, remineralization of organic matter and the presence of additional sources of DFe balanced the effect of DFe scavenging. At greater depth (>2000 m), scavenging of Fe was the main process resulting in a scavenged type distribution, as shown by a slow decrease in DFe concentration with depth (Fig. 3B). The relatively high median DFe concentration of the most shallow samples was the result of high aeolian Fe input from the Sahara and Sahel region into the western North Atlantic Ocean [73]. As a result of external sources and internal cycling, the highest natural variability in DFe concentrations were found in the upper 1000 m. Fig. 3C shows a boxplot of the DFe concentrations in the different water masses of the western Atlantic Ocean.

### The distribution of DFe in the surface ocean

**Figure 4:**
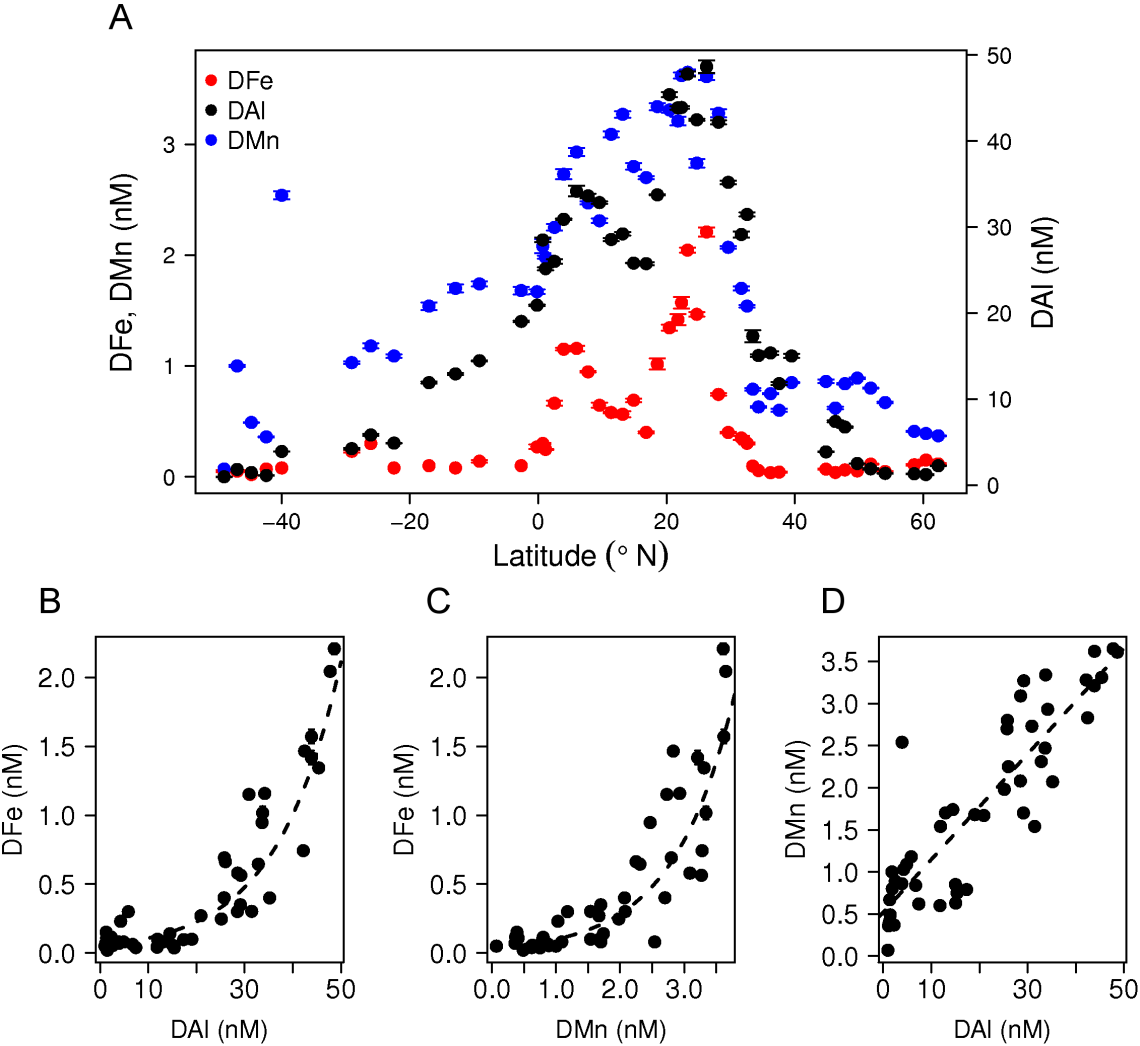
The surface distributions of DFe, DAl and DMn, and their relationships. A) Concentrations of DFe, DAl and DMn at 10 m depth along the complete western Atlantic Ocean transect. B–D) Excluding stations above the shelf and impacted by rivers, significant exponential correlations between DFe, DAl and DMn were found. The DFe correlated exponentially with B) DAl; DFe = 5.12×10^−2^ e^0.074 DAl^, R^2^ = 0.72, p = 1.9 10^−14^, n = 48 and with C) DMn; DFe = 3.43×10^−2^ e^1.06 DMn^, R^2^ = 0.78, p = 2.2 10^−14^, n = 48. DMn correlated linearly with D) DAl; DMn = 6.26×10^−2^ DAl+0.52, R^2^ = 0.78, p = 2.2 10^−14^, n = 49. Error bars represent standard deviations.

Windblown dust is the main external source of DFe to the surface of the open ocean [35] and is often seen as the most important direct source of DFe sustaining primary production. Indeed, we found a strong correlation between sea surface (10 m depth) concentrations of DFe and DAl, a proxy of dust input, for the whole western Atlantic transect. These observations confirmed that dust is an important source of DFe, especially to the subtropical surface ocean (Fig. 4A). In addition, strong correlations between surface DFe, DAl and DMn showed that dust is also an important source of bio-essential DMn to the Atlantic Ocean (Fig. 4B–D).

**Figure 5:**
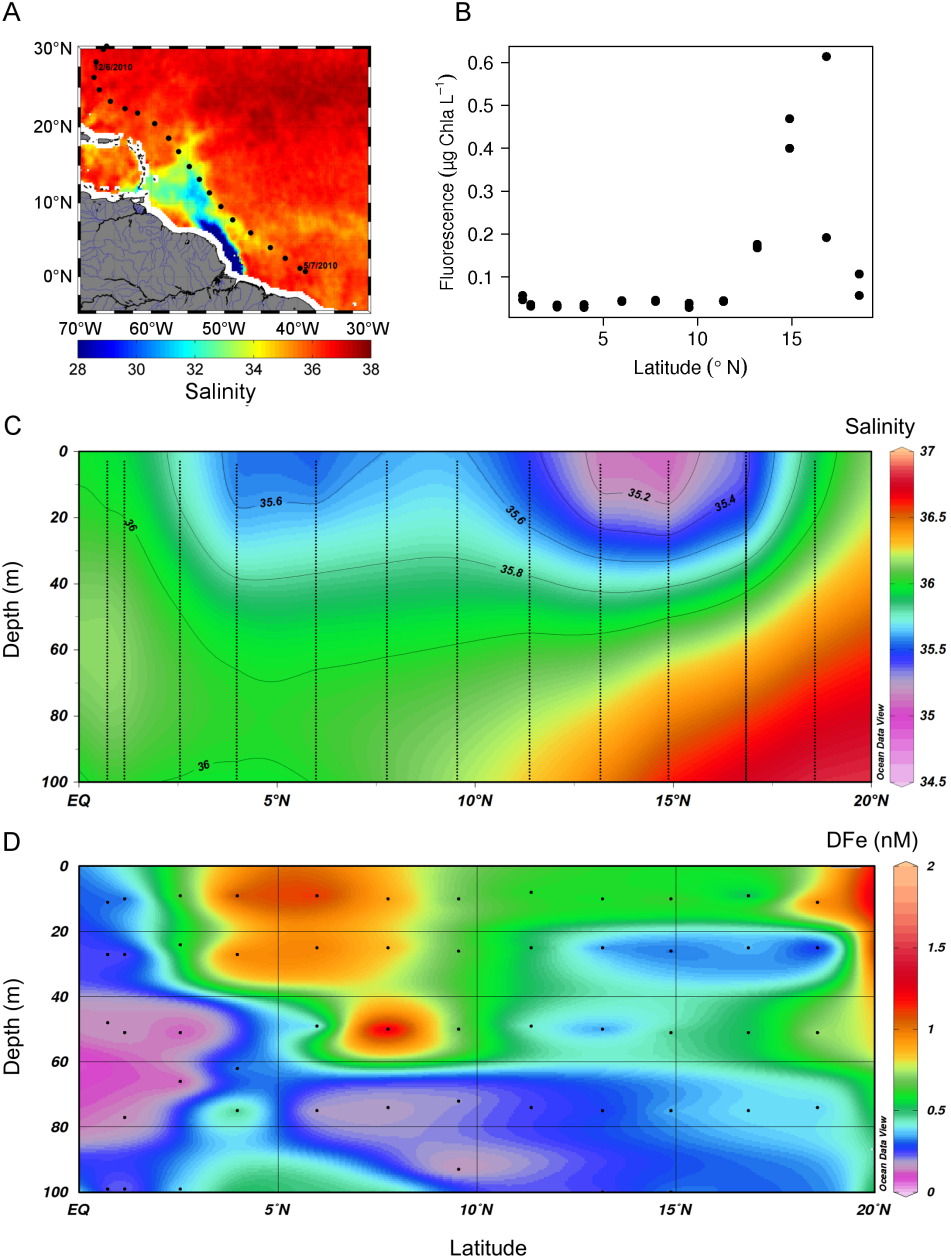
Distribution of DFe in the Amazon plume. A) The three-week composite image (12 June–5 August 2010) of sea surface salinity at 25 km resolution by the ESA SMOS satellite during the 64PE321 research cruise on the RV *Pelagia* showing the Amazon plume. Black dots represent the stations occupied during this cruise. This image was produced by the CNES/IFREMER Centre Aval de Traitement Des Données SMOS (CATDS, France). B) Fluorescence in the upper 40 m of the water column versus latitude. C) Section plot of CTD downcast salinity including salinity contour lines between the equator and 20°N showing the low saline Amazon plume. The black dots represent measurements. D) Section plot of DFe between the equator and 20°N showing the relatively high DFe concentrations in the Amazon River plume at 5°N and in comparison lower concentrations of DFe at the lower salinities of the Amazon River plume between 12–18°N.

**Figure 6:**
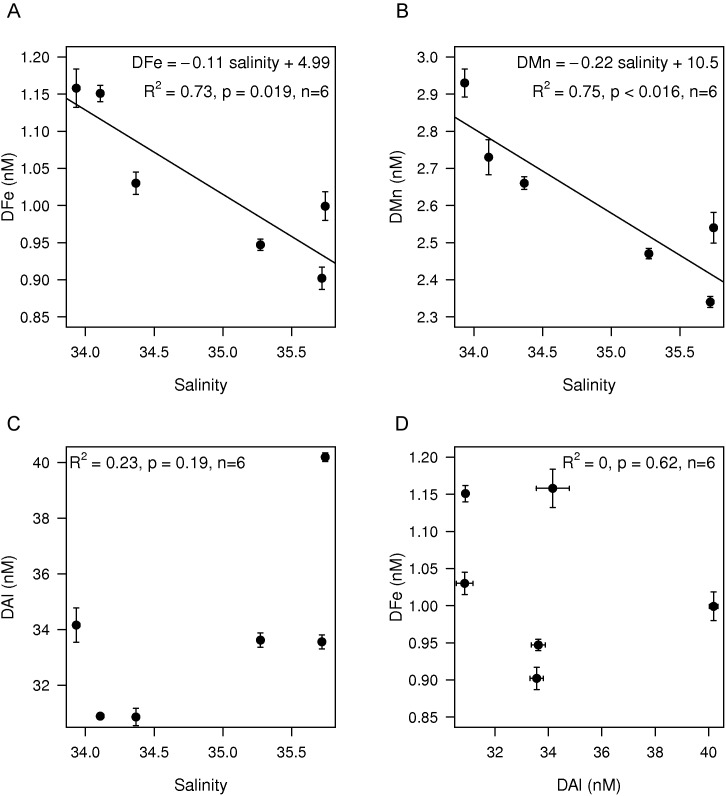
The DFe, DMn and DAl between 0–40 m depth and 3–9°N as function of salinity in the Amazon river plume. A) The significant linear correlation between DFe and salinity. B) The significant linear correlation between DMn and salinity. C) DAl as function of salinity. D) DFe as function of DAl. Error bars represent standard deviations.

Other major sources of DFe to the upper ocean came from off-shelf lateral transport and the Amazon and Rio de la Plata rivers. The Amazon River plume extended over a large area from the mouth of the Amazon at 0° to 20°N (Fig. 5A) and relatively low salinities were measured down to depths of 40 m (Fig. 5C). Within the Amazon River plume enhanced DFe concentrations of 1.03±0.11 nM (n = 6, s.d.) were measured in the upper 40 m around 5°N (Fig. 5D). Significant linear relationships of DFe and DMn with salinity, but not with DAl, indicated that at these stations between 3° and 9°N, the Amazon was the main source of DFe and DMn instead of dust (Fig. 6). Even lower salinities existed between 12° and 18°N. Surprisingly, DFe concentrations here were with 0.46±0.16 nM lower than at 5°N although still enhanced compared to background concentrations. High surface fluorescence, i.e. chlorophyll, around 15°N explained that part of the DFe was removed by biological uptake (Fig. 5B). Our study confirmed that the Amazon is an important source of Fe available for biological uptake [74].

**Figure 7:**
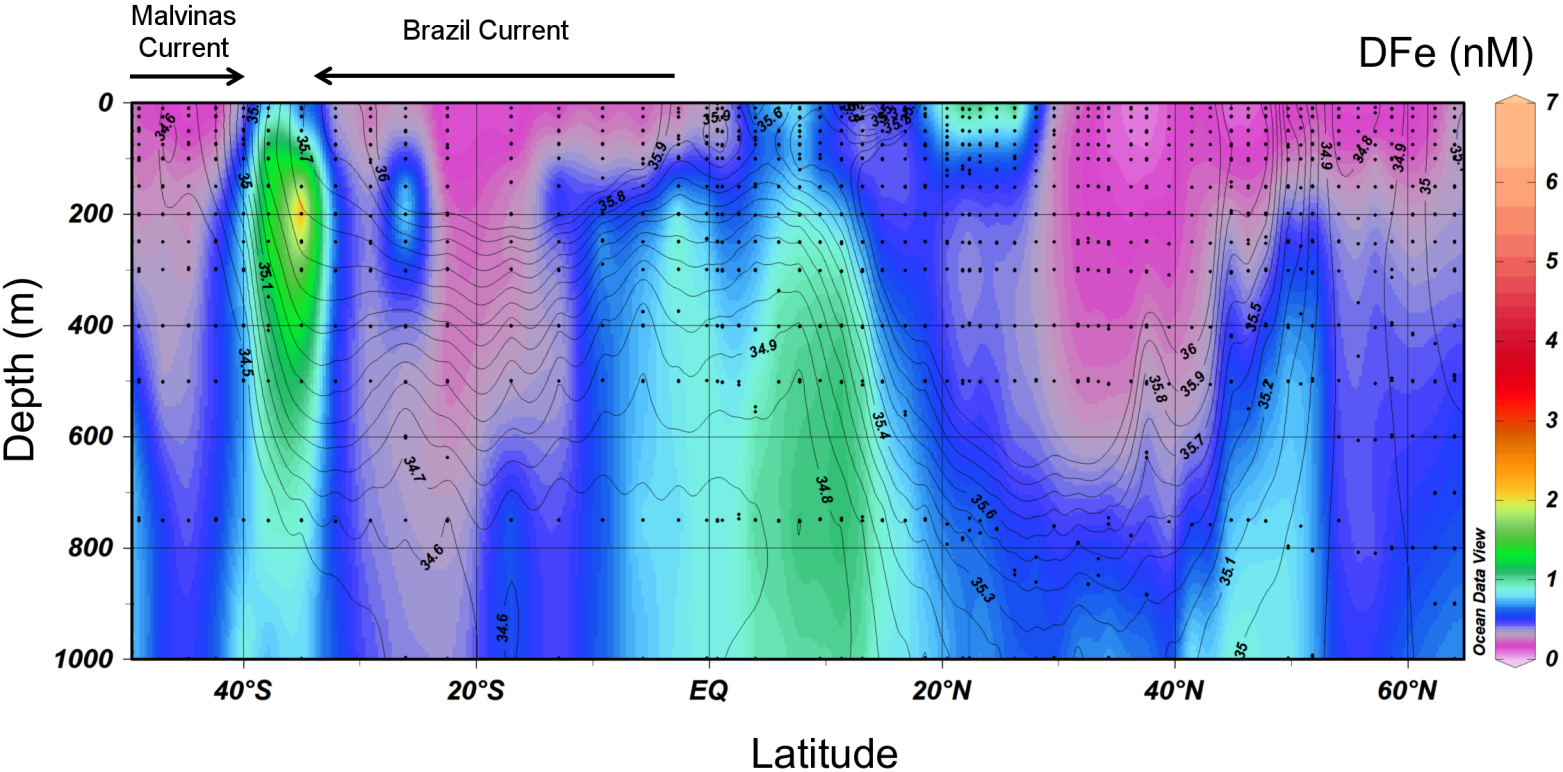
Distribution of DFe in the upper 1000 Salinity is depicted as contour lines. A steep gradient in salinity in the first 800°S and 40°S indicates the subtropical shelf front (STSF) formed by the colliding of the southward flowing Brazil Current and the northward flowing Malvinas Current. Note that the color scale is between 0–6 nM in this section plot.

**Figure 8:**
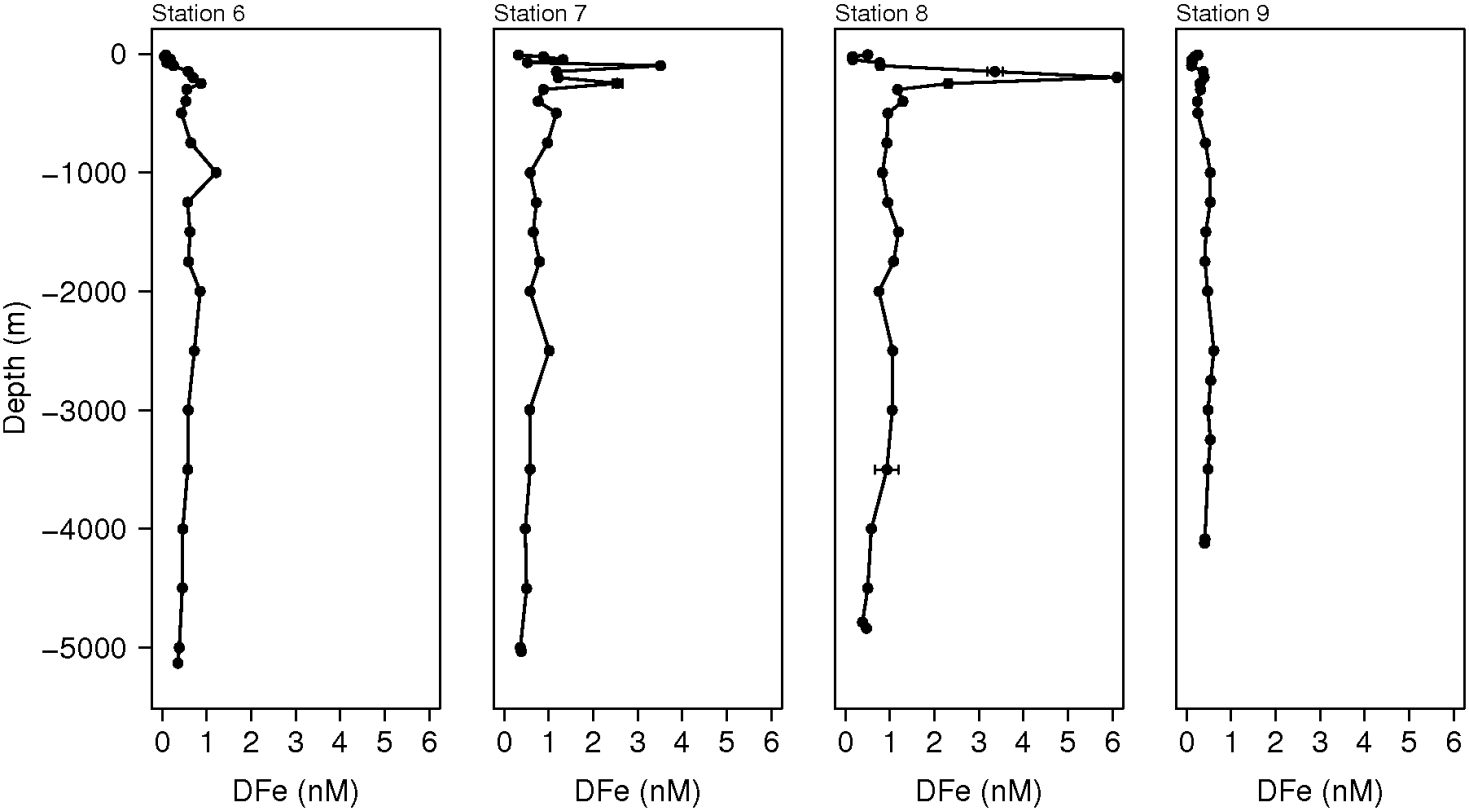
The DFe depth profiles in the subtropical shelf front between 30°S and 40°S. Full depth profiles of DFe measured during cruise 74JC057 with the RRS *James Cook* at station 6 (39°58′05″S, 42°27′32″W), station 7 (37°50′33″S, 41°07′57″W), station 8 (35°00′36″S, 39°26′21″W) and station 9 (32°05′16″S, 37°27′41″W). High DFe concentrations were found in the upper 400 m at station 7 and 8 indicating a lateral supply and partial dissolution of particulate Fe by the subtropical shelf front resulting in enhanced concentrations of DFe. Error bars represent standard deviations.

In the Southwest Atlantic, high DFe concentrations of up to 6.1 nM were observed in the upper 800 m between 35° and 40°S. These were associated with the SubTropical Shelf Front (STSF) that is formed by the southward flowing Brazil Current and the northward flowing Malvinas Current [75] (Fig. 7 and 8). The STSF extends from the shelf at 33°S in the direction of this transect at 40°S and is located in front of the Rio de la Plata at 35°S. The high DFe concentrations were not associated with a minimum in salinity indicating that the high DFe concentrations were not part of the Rio de la Plata river plume. Most likely these high DFe concentrations were the result of DFe enrichment due to offshore export of Brazilian shelf waters [75] or Fe dissolution from laterally supplied particulate iron originating from the Brazilian shelf and/or the Rio de la Plata (Fig. 7). These results are similar to findings from the North Pacific Ocean [47], [48] and the Amundsen Sea [53] where lateral transport of particles released Fe to the dissolved phase fueling phytoplankton blooms in an otherwise Fe-limited region. Lateral transport of DFe is also an important source of DFe to open ocean environments [44], [46], [52]. It is unlikely that the high DFe concentrations here were the result of Patagonian dust input [76]. The DFe concentrations in the surface ocean under the Saharan dust plume were a factor 3 lower than the high DFe concentrations found in the STSF. In addition, high DFe concentrations under the atmospheric Saharan dust plume showed maximum concentrations right at the surface while the DFe maximum in the STSF occurred at a depth of 200 m. Although enhanced concentrations of the dust proxies ^232^Th, DAl and dissolved Titanium (DTi) were found in the upper 500 m between 30° and 40°S [77], [78], [79], their concentrations did not correlate with the enhanced DFe concentrations. In addition, the enhanced DFe concentrations were much more localized compared to the distribution of these dust proxies.

### The distribution of DFe in the deep ocean

**Figure 9:**
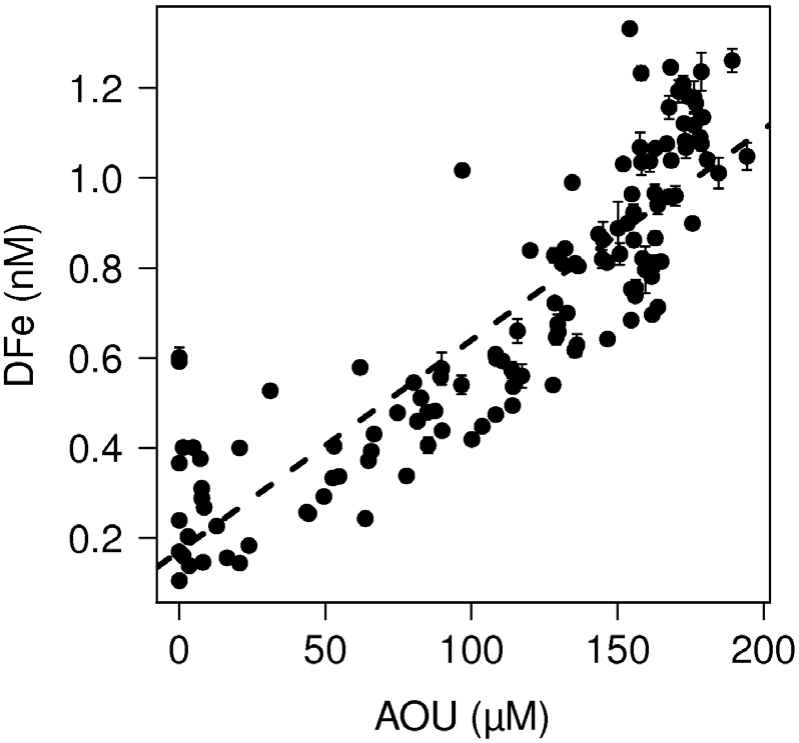
The DFe concentration as function of the Apparent Oxygen Utilization (AOU). There is a strong linear correlation between the DFe concentration and the AOU in the oxygen minimum zone between the equator and 20°N at a depth of 70–1100 m: DFe = 4.7×10^−3^ AOU+0.17, R^2^ = 0.77, p = 2.2×10^−16^, n = 129. Error bars represent standard deviations.

High concentrations of up to 1.33 nM DFe were found in the subsurface waters of the upper 70–1000 m between 0° and 20°N. The significant relationship between DFe and Apparent Oxygen Utilization (AOU) showed that remineralization of sinking organic matter was the source of these high DFe concentrations (Fig. 9). Assuming that the DFe concentrations in the OMZ related quantitatively to organic matter remineralization without being biased by scavenging, the Fe requirements of the gross phytoplankton community in surface waters above this OMZ expressed as an Fe:C ratio after conversion of AOU to remineralized carbon (C:AOU of 1.39 [80]), was 3.4×10^−6 µ^mol:mol. The average Fe:C ratio in the western North Atlantic OMZ was similar to the average Fe:C ratio of 4×10^−6^ found in the eastern North Atlantic OMZ [46].

The OMZ in the western Atlantic Ocean is the likely result of the combination of local primary production followed by decay of the sinking organic matter as well as westward transport of similar low-oxygen-high-DFe waters by the North Equatorial Counter Current from a well-known OMZ south of the Cape Verde Islands [81]. Similarly, high DFe concentrations associated with low oxygen concentrations have been reported in the upper 1000 m of the NE Atlantic between 0° and 20°N along 26–30°W [46], [82], and now even further west [83]. In summary, our observations in combination with previous studies showed high remineralization of DFe in the OMZ across the whole width of the central Atlantic Ocean.

Enhanced DFe concentrations of up to 0.99 nM were encountered between 32° and 52°N in the 1000–2000 m depth range around the Grand Banks and towards Bermuda (Fig. 3A). These enhanced DFe concentrations were measured in the LSW and uLSW around the Grand Banks and in the NADWu flowing southwards to Bermuda. The LSW flowing northwards along Greenland did not have enhanced DFe concentrations. Possibly, the southwards flowing LSW and uLSW may have picked up DFe and Fe containing particles from melting sea ice and/or the Canadian or Greenland shelf. The NEADW also contained enhanced DFe concentrations relative to surrounding waters between 57° and 65°N over the 1000–3000 m depth range (Fig. 3A, C) and forms therefore a source of DFe to the NADW.

In the deep ocean, high DFe concentrations in the range 0.8–1.55 nM coinciding with enhanced DMn concentrations (0.13–0.56 nM) were found between 2000 and 3000 m in a region from 18°S to just north of the equator, a 2100 km long section of the West Atlantic transect (Fig. 3A). High DFe coinciding with enhanced DMn confirmed that this part of the North Atlantic Deep Water (NADW) was influenced by hydrothermal vents [56], [57], [58]. Such a large hydrothermal plume enhanced in DFe has recently also been discovered in the Indian Ocean [55]. The relatively low concentrations of DMn compared to DFe is the results of a more rapid loss of Mn compared to Fe with increasing distance from the hydrothermal source as has been observed in a hydrothermal plume in the Arctic Ocean [56] and is explained by the presence of DFe in stabilized chemical forms as organic complexes or as colloids [54], [84], [85].

Westward transport of high concentrations of primordial ^3^He along 11°S from the Mid-Atlantic Ridge (MAR) [86] suggests that the plume we encountered originated from hydrothermal vents situated to the east of our section at the MAR south of the equator. Indeed, Saito et al. (2013) [60] described a hydrothermal plume with enhanced DFe between 2000–3000 m from the MAR that crossed our transect just to the south (11°S) from where we measured the highest values for DFe. However, from 11°S, the hydrothermal plume that we described spreads about 1200 km further north and 900 km further south along the path of the NADW. There are three known hydrothermal fields, the Turtle Pits field, the Nibelungen field and the Lilliput field [87], [88], [89] between 4° and 10°S along the MAR. Based on the depth of the different hydrothermal fields and the distance, Saito et al. (2013) reasoned that the hydrothermal plume they encountered originated from the Nibelungen field 1700 km to the east. The closest discovered hydrothermal field at the MAR north of the equator is the Ashadze field (12°58′N, 44°51′W) [90] at 2000 km north of where we encountered the hydrothermal plume. The Ashadze and the Nibelungen hydrothermal fields are equally likely to be the origin of the hydrothermal plume that we encountered. Moreover, the highest DFe concentrations were measured at the northern part of the transect, close to the equator at a distance of only 300–400 km from the MAR. Undiscovered hydrothermal vent fields could also be responsible for the hydrothermal plume that we encountered.

### Fe as limiting nutrient

**Figure 10:**
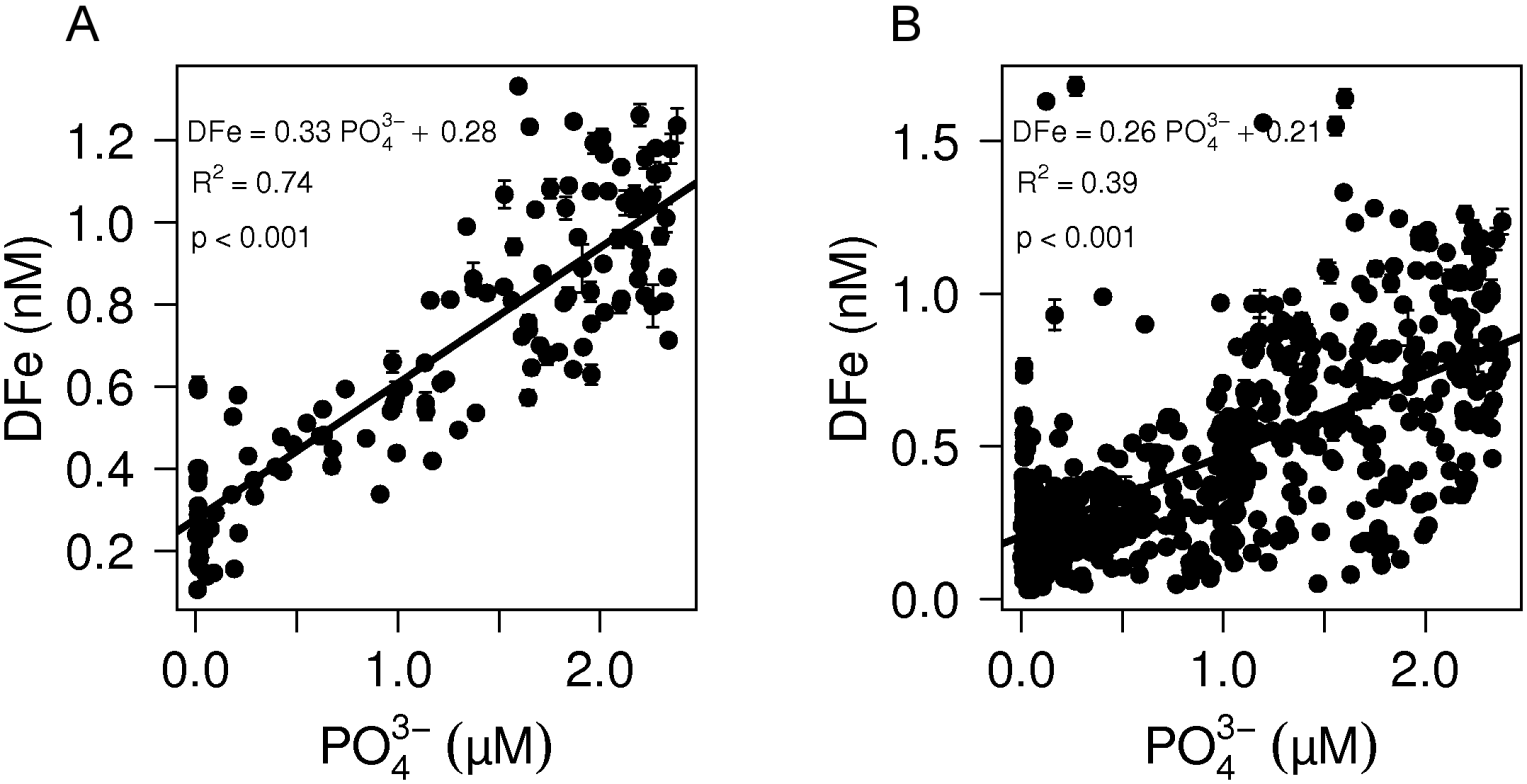
The DFe concentration as function of PO_4_^3−^. A**)** There is a strong linear correlation between the DFe concentration and the PO_4_^3−^ in the oxygen minimum zone between the equator and 20°N at a depth of 70–1100 m, and B) in comparison the correlation between DFe and PO_4_^3−^ over the whole West Atlantic between 50–1000 m depth where remineralization of organic matter is the dominant source of DFe. The high DFe data in the STSF were left out. Error bars represent standard deviations.

To assess the surface areas and deep water masses where DFe concentrations potentially limit phytoplankton growth (when reaching the surface) we subtracted the contribution of organic matter remineralization to the dissolved Fe pool using the tracer Fe*. Fe* is defined as Fe* = [DFe]−R_Fe:P_×[PO_4_^3−^] [91], where R_Fe:P_ is the average biological uptake ratio of Fe over PO_4_^3−^. A large variation in R_Fe:P_ exists depending on the methodology to determine R_Fe:P_ but also depending on regional differences in DFe concentration and differences in phytoplankton community structure and may vary between different oceans [92]. Although an average uptake ratio of 0.47 mmol:mol Fe:PO_4_^3−^ has been used before [91], [93], [94], we used an average uptake ratio of 0.33 mmol:mol DFe:PO_4_^3−^ as found in the West Atlantic OMZ (Fig. 10A). This value was close to the DFe:PO_4_^3−^ stochiometry of 0.26 mmol:mol as measured between 50 and 1000 m depth along the whole West Atlantic transect. It is in this depth range where most Fe is released in the dissolved phase during organic matter remineralization (Fig. 10B). We used the slope of 0.33 mmol:mol Fe:PO_4_^3−^ to calculate Fe* because i) the effect of organic matter remineralization is strongest in the OMZ, and ii) the strong linear relationship suggests that that this slope represents the uptake stochiometry of DFe over PO_4_^3−^ by the phytoplankton community overlying this OMZ without being strongly influenced by Fe scavenging. An excess of organic Fe-binding ligands of 0.74±0.54 nE of M Fe (n = 23) in the OMZ confirmed that scavenging of DFe was low due to its stabilization by organic Fe-binding ligands in the OMZ [29], [30], [31].

Plotting DFe versus PO_4_^3−^ in the OMZ as well as along the West Atlantic transect resulted in a positive intercept with the DFe-axis of 0.28 nM and 0.21 nM DFe, respectively (Fig. 10). This excess of DFe after full biological utilization of PO_4_^3−^ is unrelated to the biological recycling of DFe and is therefore the net result of external input and transport of Fe on the one hand and scavenging of Fe on the other hand.

**Figure 11:**
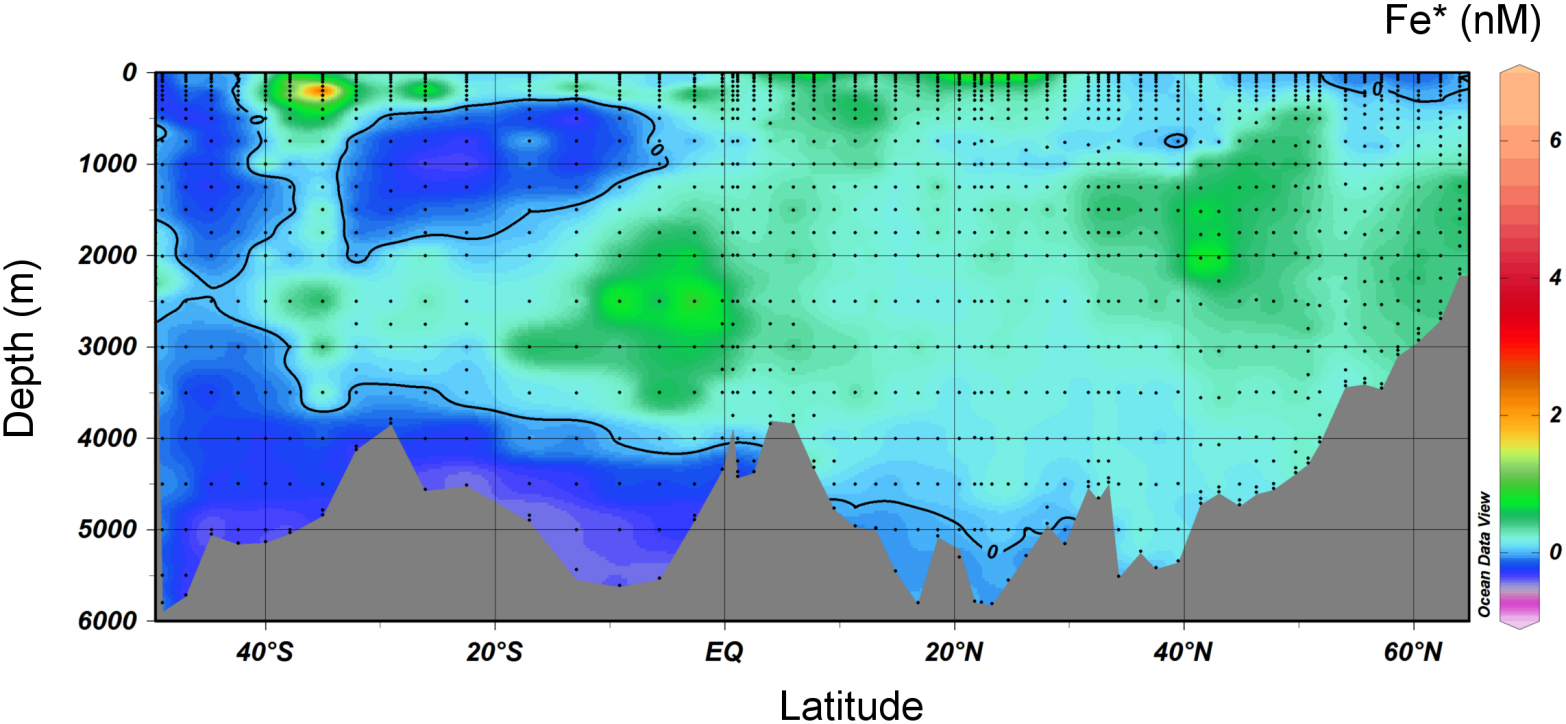
Section plot of the Fe* tracer in the western Atlantic Ocean. Negative values indicate potential growth limiting DFe concentrations and positive values indicate an excess in DFe after complete biological uptake of PO_4_^3−^. A contour line of 0 separates areas of negative Fe* from areas with positive Fe*.

Assuming an average uptake ratio R_Fe:P_ of 0.33, negative values of Fe* indicate potentially growth limiting concentrations of DFe while positive values of Fe* indicate an excess of DFe relative to the uptake of PO_4_^3−^. A section plot of Fe* shows potentially limiting concentrations of Fe (Fe* <0) south of 47°S and north of 52°N consisting of 15% of the surface waters of the western Atlantic transect (Fig. 11). Indeed, seasonal Fe stress of phytoplankton has been observed in the western Irminger basin north of 52°N [95]. In the deep ocean, negative values of Fe* were found in the Antarctic Bottom Water (AABW) and the Antarctic Intermediate Water (AAIW). Positive Fe* was found in most of the NADW. Modeling suggests that high concentrations of Fe* enter the NADW after northward transport of aeolian derived Fe with the western boundary current [91]. This may explain the enhanced DFe concentrations found in the NEADW. We now showed that additional DFe also enters the NADW with the LSW and uLSW in the North Atlantic Ocean. We also showed that hydrothermal DFe input is a source of Fe that enhances the concentrations of Fe* in the NADW in the southern hemisphere.

High dust input and transport of Fe by the STSF to the open ocean are the most important external sources of DFe to the surface of our transect resulting in high surface Fe* concentrations between 0° and 30°N, and 25° and 40°S, respectively. These regions with high external Fe input coincide with, and therefore are pivotal to facilitate, high nitrogen fixation in the North Atlantic Ocean [12] and high primary productivity in the South Atlantic STSF during the austral summer [96].

## Conclusions

The DFe distribution over the full 4–6 km water depth along the 17500 km long transect in the West Atlantic Ocean was, as indicated by the hybrid type depth distribution of the median DFe concentration, dominated by vertical processes. This hybrid type depth profile showed that, in the upper 1000 m, processes that dominated the DFe distribution were biological uptake in the surface followed by recycling of Fe via the remineralization of sinking organic matter at depth. Between 1000 and 2000 m the recycling of Fe together with other sources of Fe balanced the scavenging of DFe. Below 2000 m it was the scavenging of DFe that was the important sink of DFe.

The distribution of DFe in the West Atlantic Ocean was also influenced by external sources of DFe. Important external sources of DFe to the surface West Atlantic Ocean were Saharan dust and the input of DFe from the Amazon River. Lateral transport of Fe in dissolved or particulate state from the shelf could be an important process explaining enhanced DFe concentrations in the STSF and between 32 and 52°N in the uLSW, the LSW and NADWu. In the deep West Atlantic Ocean, south of the equator, we found a large plume of enhanced DFe from a hydrothermal vent.

Finally, the section plot of Fe* showed that the Atlantic Ocean via the NADW as part of the ocean conveyor belt is an important source of Fe in excess of phosphate for biological production in the global oceans.
